# Label-free analytical characterization of brazzein produced with the filamentous fungus *Trichoderma reesei*

**DOI:** 10.3389/fbioe.2025.1688495

**Published:** 2025-12-12

**Authors:** Dominik Mojzita, Nina Aro, Juha Kontturi, Martin Kögler, Emilia Nordlund, Waltteri Hosia, Marco G. Casteleijn

**Affiliations:** 1 VTT Technical Research Centre Finland, Espoo, Finland; 2 VTT Technical Research Centre Finland, Oulu, Finland

**Keywords:** brazzein, Trichoderma, Raman spectroscopy, fungal expression system, sweet protein

## Abstract

Brazzein, a small, pH-stable, and heat-stable sweet protein, is a promising alternative to sucrose because of its extreme sweetness and stability. However, large-scale production through traditional extraction methods from plant sources is not economically feasible. To this end, the current study explores the production of recombinant brazzein in the filamentous fungus *Trichoderma reesei* using a synthetic expression system (SES) and the native fungal *cbh1* promoter. Both methods successfully expressed brazzein in 24-well liquid cultures, with the SES-system achieving a production level of ∼1.3 g/L in bioreactor cultures. The protein was purified and characterized using SDS-PAGE, liquid chromatography high-content mass spectroscopy (LC-HCMS), Timegated™ Raman spectroscopy, dynamic light scattering (DLS), and nanoscale differential scanning fluorimetry (nanoDSF). The results confirmed that the correct folding and stability of brazzein attributed to its robust internal structure, demonstrating the potential of *T. reesei* as an efficient host for producing this sweet protein.

## 1 Introduction

Sweet proteins are a group of structurally diverse proteins with no shared sequence similarity. They differ in molecular size and sweetness potency, but they are generally small (6.4 kDa–22.2 kDa) and are 500–4,000 times sweeter than sucrose (w/w) (Leone et al., 2016; Picone and Temussi, 2012). To date, eight sweet proteins have been identified: brazzein (Ming and Hellekant, 1994), thaumatin (van der Wel et al., 1975), monellin (INGLETT and MAY, 1969), miraculin (Takahashi et al., 1990), pentadin (Faus, 2000), mabinlin (Liu et al., 1993), curculin (Harada et al., 1994), and lysozyme (Maehashi and Udaka, 1998). Most of these proteins have been purified from tropical plants of South Asia or Africa, except lysozyme which is derived from egg white.

Brazzein is the smallest naturally occurring sweet protein, measuring only 6.4 kDa in size. The monomer consists of 54 amino acids and was first isolated from the fruit of *Pentadiplandra brazzeana*, also known as oubli, found in West Africa. Brazzein, in its powder form, is 2,000 times sweeter than a 2% (w/v) sucrose solution (Izawa et al., 1996). Whereas brazzein is derived from the fresh form of the fruit, another sweet protein, pentadin, is extracted from the heat-dried form of the fruit. Pentadin is the cross-linked, non-native form of brazzein, with twice the molecular weight and considerably reduced sweetness compared to brazzein (Assadi-Porter et al., 2000). Extracted brazzein is heterogeneous and consists of two structurally different forms. The majority of brazzein has a pyroglutamic acid (pGlu) at the amino-terminal residue; the minor version has the same sequence but lacks the amino-terminal residue (*des*-pGlu1-brazzein) (Ming and Hellekant, 1994). The sweetness of *des*-pGlu1-brazzein is approximately twice that of pGlu1-brazzein (Assadi-Porter et al., 2000). Correct folding of this small protein is crucial for its functionality and binding to taste receptors (Temussi, 2011; Zhao et al., 2021).

**FIGURE 1:**
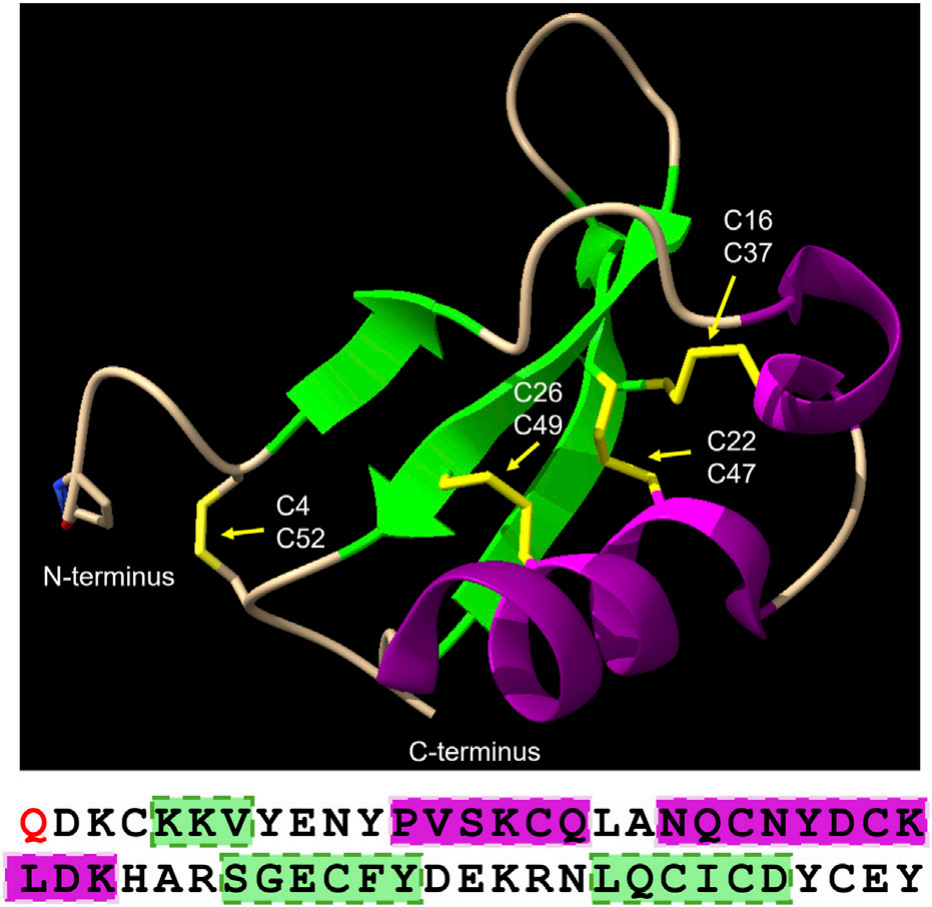
Representation of the brazzein protein structure (based on the PDB file 4HE7; image created with ChimeraX v1.10). Green ribboned arrows highlight the β-strands, and magenta highlights the α-helical structures. Shown in yellow, and also indicated by the yellow arrows, are the four disulfide bonds contributing to brazzein’s thermostability. The N-terminal pyroglutamic acid is shown as a stick model. The amino acid sequence is listed directly below the image. The red *Q* indicates the N-terminal pyroglutamic acid, and the green and magenta boxes correspond to the β-sheet and α-helical structures in the structural image, respectively.

Brazzein is highly stable; it remains folded up to ∼85 °C and retains some of its sweetness at above 80 °C, but only ∼20% at 100 °C (Zuo et al., 2024). ^1^H NMR spectroscopy studies have revealed that the structure of brazzein (Figure 1) is compact, containing two short α-helices and three β-sheets linked together by four disulfide bridges and loops, which explains its thermal and pH stability (Caldwell et al., 1998; Ming and Hellekant, 1994). The brazzein structure also contains three loops (loop I, residues 9–19; loop II, residues 30–33; and loop III, residues 38–45). These loops and the N- and C-terminal regions of the protein have been identified as responsible for the sweetness of brazzein since they are involved in the interactions between brazzein and the sweet-tasting receptor (Assadi-Porter et al., 2010). Brazzein interacts with the G protein-coupled T1R2–T1R3 sweet receptor in a specific binding mode, as described earlier (Moralejo et al., 2001; Temussi, 2011; Tancredi et al., 2004; Yamamoto et al., 2020; Zhao et al., 2021).

Sweet proteins are potential low-calorie substitutes for sugar and traditional sweeteners in the food and the pharmaceutical industries. However, major hurdles include limited extraction from fruits and low recombinant yields. The concentration of brazzein in ripe fruits is approximately 0.02%–0.5% by weight (Hellekant and Danilova, 2005), whereas the typical yields in precision fermentation in yeasts and bacteria (Kaplan Türköz et al., 2022), transgenic maize or carrots (Lamphear et al., 2005; Han et al., 2022), or the milk of transgenic mice (Yan et al., 2013) have ranged from micrograms to 0.345 g/L. The loss of sweetness has also been observed in recombinant protein samples (Assadi-Porter et al., 2000; Berlec et al., 2006), indicative of protein misfolding.

The filamentous fungus *Trichoderma reesei* has been used for the production of many different proteins, such as cellulases, amylases, xylanases, and proteases (Druzhinina and Kubicek, 2017; Mäntylä et al., 2007; Miettinen-Oinonen and Suominen, 2002); interferon alpha-2b (Landowski et al., 2016); and food proteins (Aro et al., 2023). Due to its ability to secrete proteins at yields as high as 100 g/L (Cherry and Fidantsef, 2003) and its GRAS status, *Trichoderma* is widely used in industrial processes.

Here, we utilized engineered *T. reesei* QM9414 strains for brazzein production in 24-well plates (batch phase) and in a 250-mL fed-batch fermentation system. Product identity and folding were assessed using liquid chromatography high-content mass spectroscopy (LC-HCMS), circular dichroism (CD) spectroscopy, nanoscale differential scanning fluorimetry (nanoDSF), and Timegated™ Raman spectroscopy. Long-term stability after 24 months at 4 °C–8 °C was evaluated using dynamic light scattering (DLS).

## 2 Materials and methods

All chemicals were obtained from Sigma-Aldrich (United States), unless otherwise specified.

### 2.1 Strains

*Escherichia coli* DH5α (Life Technologies) was used for the propagation of the plasmids. *T. reesei* strain M1908 was obtained from the strain developed by the VTT Technical Research Centre of Finland. The M1908 strain was generated from the M124 strain by sequentially deleting 11 secreted proteases as described before (Aro et al., 2023). Spore suspensions were prepared by cultivating the fungus on potato-dextrose (PD) plates (BD, Sparks, Maryland, United States) for 5 days, after which the spores were harvested; suspended in buffer A containing: 0.8% NaCl, 0.025% Tween-20, and 20% glycerol; filtered through cotton; and stored at −80 °C.

### 2.2 Molecular biology

To construct brazzein expression vectors, *T. reesei* codon-optimized gene fragments encoding brazzein with either the HFBI signal sequence or the CBHI carrier were obtained from a synthetic gene provider (GenScript Biotech, the Netherlands). Both fragments were cloned into *Pac*I-linearized SES-vector pAWP145 (Aro et al., 2023) to generate expression vectors pSWP3 and pSWP4, respectively, by GenScript Biotech according to their proprietary methods (see Supplementary Figure S1). For expression from *cbh1* promoter, the carrier containing the fragment was cloned into *Pac*I-linearized pAWP128 vector to generate the expression plasmid pSWP10. The expression cassettes were liberated from the plasmids with *Pme*I restriction enzyme and purified from agarose gel.

#### 2.2.1 Trichoderma transformation

To generate brazzein production strains, the M1908 strain was transformed with pSWP3, pSWP4, and pSWP10 expression cassettes using CRISPR/Cas9 transformation (Che, 2003; Makarova et al., 2013), targeting *egl1* and *cbh1* loci as described earlier (Rantasalo et al., 2019). The primers used for targeted integration and PCR screening are listed in Supplementary Table S2. Hygromycin was used as the selection marker for transformations with the SES-expression constructs, whereas the *pyr4* selection marker was used for the transformation with the *cbh1*-promoter-containing construct.

Correct integration of the expression cassettes was assessed with PCR (3′- and 5′-ends and deletion of the *egl1* or *cbh1* ORF (Supplementary Table S2). The selected transformants were purified through single-colony plating. The best performing clones were grown on PD plates to induce spore formation. Spores were harvested and then suspended and stored in buffer A at −80 °C until further use.

#### 2.2.2 Protein expression and sample preparation

For protein expression, transformants were cultivated in 3 mL within 10 mL 24-well plates (Cytiva, United States) for 5 days (28 °C, 200 RPM) and sampled at days 3, 4, and 5. The plates were sealed with a paper seal to allow maximal oxygen transfer to the cultivation broth under constant agitation in the incubator-shaker. Cultivations were carried out either in cellulase-inducing spent grain extract-lactose (SGE-lactose) medium or glucose-containing medium (Aro et al., 2023).

Bioreactor cultivations were carried out in 250-mL Ambr reactors (Sartorius, Germany). Prior to the inoculation, cells were pre-cultivated in 250-mL Erlenmeyer flasks for 2 days (28 °C, 220 RPM) in pre-culturing glucose-containing media (Aro et al., 2023). The Ambr 250 automated high-throughput bioprocess system (Sartorius, Germany) was used for the strain behavior assessment in a fed-bath process at 28 °C of strains M3728 (pH 4.0) and M3730 (pH 4.8) using the same glucose-containing media, with foaming controlled by the addition of Dow Corning 1500 antifoam (3mL/L). Two different feeding strategies (“low feed” = 1.6 g glucose L^−1^ h^−1^ and “high feed” = 2.1 g glucose L^−1^ h^−1^) were used in two bioreactors. In addition, the cultivation conditions in the 24-well automated Ambr (Sartorius) fed-batch fermentations were pH (at pH 4.0 or pH 4.8)- and temperature (at 28 °C)-controlled. Samples were taken daily in tubes at 4 °C and filtered (Whatman GF/B) on the same day prior to SDS-PAGE analysis. Brazzein samples were purified in deionized water prior to LC-HCMS, Timegated™ spectroscopy, DLS, and nanoDSF analyses to remove impurities (at approximately 60 kDa; Supplementary Figure S2) using an ultrafiltration step (Vivaspin mwco 10 kDa; Sartorius, Germany) according to the manufacturer’s protocol. For the Timegated™ Raman spectroscopy analysis, the samples were diluted with deionized water to a concentration of 1.0 g/L. One sample was heated at 75 °C for 20 min, followed by centrifugation. For the DLS measurements, the sample was stored for 24 months at 4 °C–8 °C before analysis.

#### 2.2.3 SDS-PAGE analysis and protein quantification

SDS-PAGE analysis was carried out using 16.5% Criterion™ Tris–tricine gels that were stained with PageBlue protein staining solution (Thermo Fisher Scientific) and imaged using an Odyssey CLx imaging system instrument (LICORBio, USA). For brazzein quantification, a dilution series (0.3125 µg–5.0 µg) of recombinant brazzein with reference SKU: AA-019 (Amidebio, USA) was run along with the samples in SDS-PAGE. The protein amounts were quantified using densitometry (Bio-Rad, USA), and their images were analyzed using Image Lab software version 6.1.

### 2.3 Mass spectrometric sample preparation and analysis

Sample handling for intact mass analysis was conducted as follows: the non-heat-treated sample was centrifuged at 12,000 rpm for 20 min, and 10 µL of the supernatant was transferred to the UPLC vial and diluted with 90 µL of ultrapure water to a final concentration of 0.2 mg/mL. Heated samples were treated identically to the ambient samples, except they were first incubated for 20 min at 70 °C (i.e., heat-treated samples). Reduced samples were prepared by spiking the heat-treated samples with 150 mM DTT to a final DTT concentration of 15 mM and incubating them for 30 min at 50 °C. The reduced and urea-diluted sample (final urea concentration ∼7M) was obtained by mixing 10 µL of the reduced sample with 40 µL saturated (∼9 M) urea, and the mixture was briefly vortexed. Intact mass samples were analyzed using a UPLC-MS instrumentation Waters Synapt G2-Si-QTOF mass spectrometer with the Waters Acquity UPLC front end. The UPLC parameters were as follows:

**Table:** 

Column	Acquity UPLC® CSH C18 1.7 µm, 2.1 × 100 mm		
Column temperature (°C)	60.0		
Run time (min)	18.5		
Buffer A	0.1% formic acid in UP water		
Buffer B	Acetonitrile		

Gradient table			
Time (min)	Flow rate	%A	%B
1. Initial	0.350	98.0	2.0
2. 2.00	0.350	98.0	2.0
3. 12.00	0.350	40.0	60.0
4. 13.00	0.350	5.0	95.0
5. 15.50	0.350	5.0	95.0
6. 16.00	0.350	98.0	2.0
7. 18.50	0.350	98.0	2.0
Main mass spectrometer parameters			
m/z range	435–1,990		
Polarity	ES+		
Capillary (kV)	2.7900		
Source temperature (°C)	135		
Sampling cone (V)	43.0		
Desolvation temperature (°C)	400		
Desolvation gas flow (L/Hr)	900.0		
Mode	Mse		
Scan time (sec)	0.2		
Collision energy low (eV)	25.0		
Collision energy high (eV)	95.0		

Data processing was carried out using Waters MassLynx software, including deconvolutions with the MaxEnt 3 algorithm. Masses were calculated with the MassLynx BioLynx peptide editor based on the sequence, pQDKCKKVYENYPVSKCQLANQCNYDCKLDKHARSGECFYDEKRNLQCICDYCEY, where pQ is the N-terminal pyroglutamic acid. Theoretical monoisotopic masses for non-reduced and reduced proteins were 6476.828 Da and 6468.766 Da, respectively.

Peptide map mass spectrometric (P-MS) analysis was carried out in the Turku Proteomics Facility (University of Turku and Åbo Akademi University). Samples were cut from the SDS-PAGE gel, digested in-gel according to the standard protocol, and analyzed with LC-ESI-MS/MS using a Q Exactive HF mass spectrometer. Database searches were performed by the Mascot search engine against the brazzein protein sequence database.

Digested peptides were dissolved in 0.1% formic acid, and a measure of 200 ng of peptides from each sample was submitted to LC-ESI-MS/MS analysis. Wash and blank runs were submitted between each sample to reduce the carry-over of highly abundant peptides.

The LC-ESI-MS/MS analysis was performed on a nanoflow HPLC system (EASY-nLC 1000, Thermo Fisher Scientific) coupled to the Q Exactive HF mass spectrometer (Thermo Fisher Scientific, Germany) equipped with a nano-electrospray ionization source. Peptides were first loaded on a trapping column and subsequently separated inline on a 15-cm C18 column (75 μm × 15 cm, ReproSil-Pur 5 μm 200 Å C18-AQ) (Dr. Maisch HPLC GmbH, Ammerbuch-Entringen, Germany). The mobile phase consisted of water with 0.1% formic acid (solvent A) or acetonitrile/water (80:20 (v/v)) with 0.1% formic acid (solvent B). A 30-min gradient (from 8% to 43% solvent B, following a wash stage at 100% solvent B) was used to elute peptides.

MS data were acquired automatically using Thermo Xcalibur 4.1 software (Thermo Fisher Scientific). An information-dependent acquisition method consisted of an Orbitrap MS survey scan of mass range 300–2,000 m/z followed by HCD fragmentation.

Data files were searched for protein identification using Proteome Discoverer 2.3 software (Thermo Fisher Scientific, Germany) connected to an in-house server running Mascot 2.6.1 software (Matrix Science). Data from samples 1 and 2 were searched against a custom-made database containing defensin-like protein P56552 of “*Pentadiplandra brazzeana*” (downloaded from UniProt 19.11.2019).

### 2.4 Dynamic light scattering (DLS)

DLS measurements of brazzein in water (2 mg/mL) were performed using a Malvern Zetasizer Nano-ZS90 at a 173° backscattering angle equipped with a 633-nm He−Ne laser at a controlled temperature of 20 °C ± 1 °C at the start of thermal ramping, which was then increased to 92 °C at a ramp rate of 0.77 °C/min. The acquisition time was 5 s, and five acquisitions were averaged per measurement.

### 2.5 Circular dichroism (CD)

A Chirascan^TM^ CD spectrometer equipped with a temperature-controlled unit (at 22 °C ± 1 °C and at 94 °C ± 1 °C) was used to collect spectra using a QS quartz cuvette with 1 mm path length. For brazzein protein in water (0.1 mg/mL), data acquisition was performed across the wavelength range of 180–280 nm, with a 1-nm bandwidth, 1-nm step size, and an averaging time of 0.5 s. For each sample, measurements were repeated five times; the spectra were averaged and smoothed. The spectra were analyzed using BeStSel v1.3.230210.

### 2.6 Timegated™ surface-enhanced Raman spectroscopy

Both samples (one at room temperature and one that was treated at 75 °C for 20 min) were analyzed using a wavelength-calibrated commercial Timegated™ Raman spectrometer (PicoRaman from Timegate Instruments, Oulu, Finland) using a pulsed laser with λ_exc_ = 532 nm excitation wavelength, 100 mW laser power, 100 ps pulse length, and approximately 100 kHz repetition rate. The TimegatedTM Raman spectroscopy system encompassed a temperature-stabilized CMOS SPAD array (8 × 768 pixels single-photon counting) detector with a spectral resolution of 5 cm^−1^ and temporal resolution of 100 ps. The TGRS was coupled to a conventional non-immersion Raman probe equipped with a quartz window (B&W Tek, BAC102, Metrohm, Herisau, Switzerland) and a working distance of 5.4 mm. The raw data were post-processed using Timegated^TM^ software. In addition, the spectra were baseline-corrected and smoothed using an SG (Savitzky–Golay) algorithm with the second polynomial order and five-point window using the software package OriginPro 2021b (64-bit) SR2 version 9.8.5.212 (OriginLab Corporation, MA, USA).

### 2.7 Nanoscale differential scanning fluorimetry (nanoDSF)

Protein thermal stability was analyzed using a Prometheus NT.48 instrument (NanoTemper Technologies). Capillaries were filled with 10 μL of protein solution at the original concentration (1.97 mg/mL) and at a 1:3 or 1:9 dilution in dH_2_O. The temperature was increased from 20 °C to 95 °C at a ramp rate of 1 °C/min. The excitation wavelength was 280 nm, and the ratio of emission intensities (Em_350nm_/Em_330nm_) was recorded. The fluorescence intensity ratio and its first derivative were calculated using PR.ThermControl (NanoTemper Technologies).

## 3 Results

The parental *T. reesei* strain M1908 transformed with the SES promoter constructs (pSWP3 and pSWP4) or the *cbh1* promoter construct (pSWP10) exhibited brazzein production profiles when initially assessed in 24-well plate cultures. Cultivation in a glucose-based medium supported baseline expression, whereas the lactose-containing, cellulase-inducing medium resulted in higher production levels across the tested strategies. In glucose cultivations, the highest brazzein production was observed from the transformant pSWP4-1, whereas in lactose cultivations the highest brazzein production was seen from the transformants pSWP4-1 and pSWP10-1 (Supplementary Figure S2). These transformants along with the transformant pSWP3-2 were purified through single-colony plating, subsequently resulting in the following production strains: (a) M1908 + pSWP3 = M3727; (b) M1908 + pSWP4 = M3728; and (c) M1908 + PSWP10 = M3730.

**FIGURE 2:**
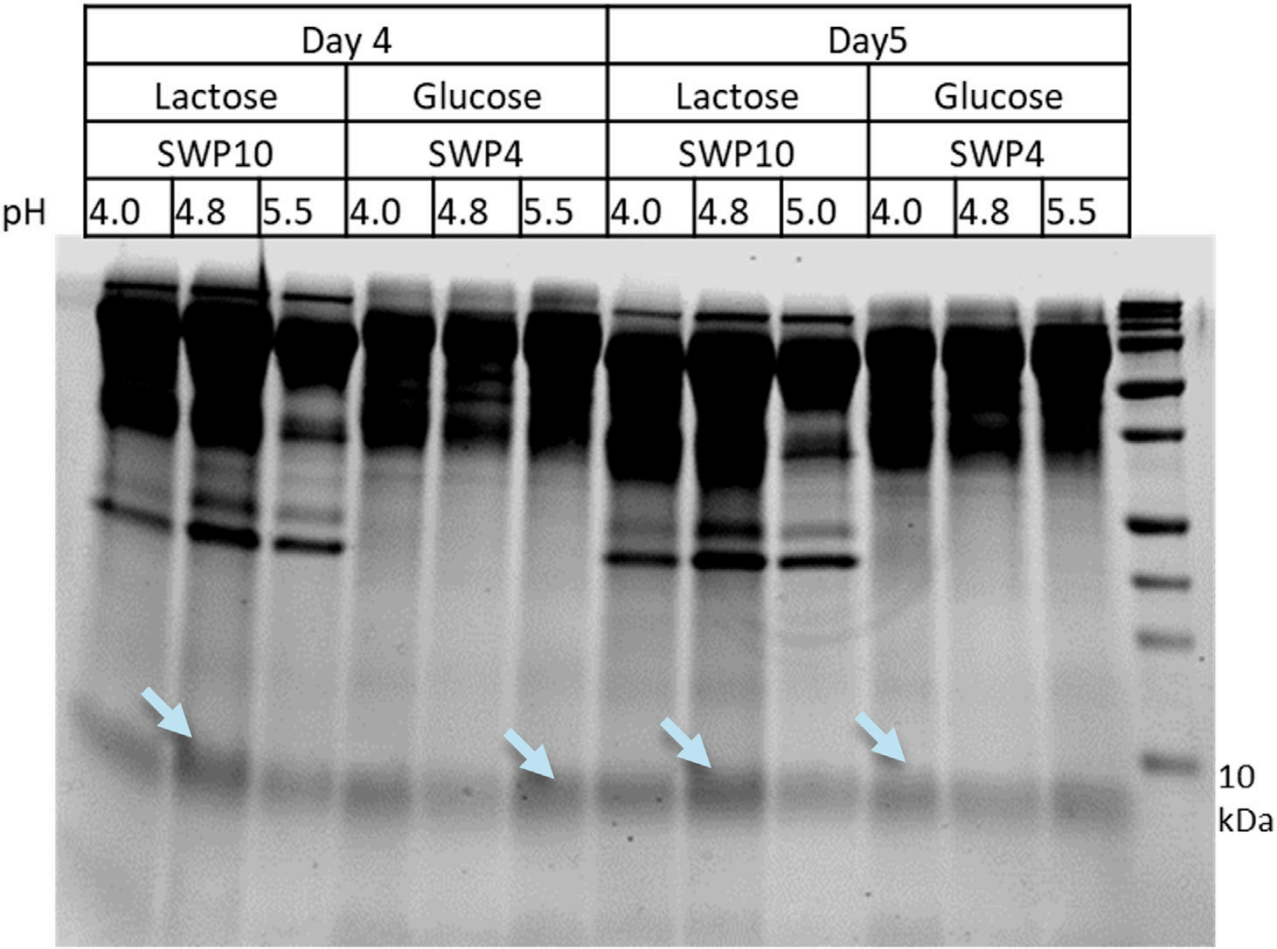
SDS-PAGE analysis of brazzein secretion optimization screening. For comparison, the final 2 days of cultivation are shown (days 4 and 5), and the M1908 strains transformed with plasmids pSWP4 or pSWP10 were either cultivated on lactose- or glucose-containing medium (either pH 4.0, 4.8, or 5.5) corresponding to six different cultivations. Bands marked by light blue arrows were cut for MS analysis confirming the target protein brazzein (data not shown).

Productions strains M3727, M3728, and M3730 were cultivated in both glucose and lactose media across three pH values to assess the effects on brazzein titers (Figure 2). For the M3730 strain, a pH of 4.8 yielded the best outcome for brazzein production using lactose media, whereas for M3728 in glucose media, the pH had no influence on the production levels.

**FIGURE 3:**
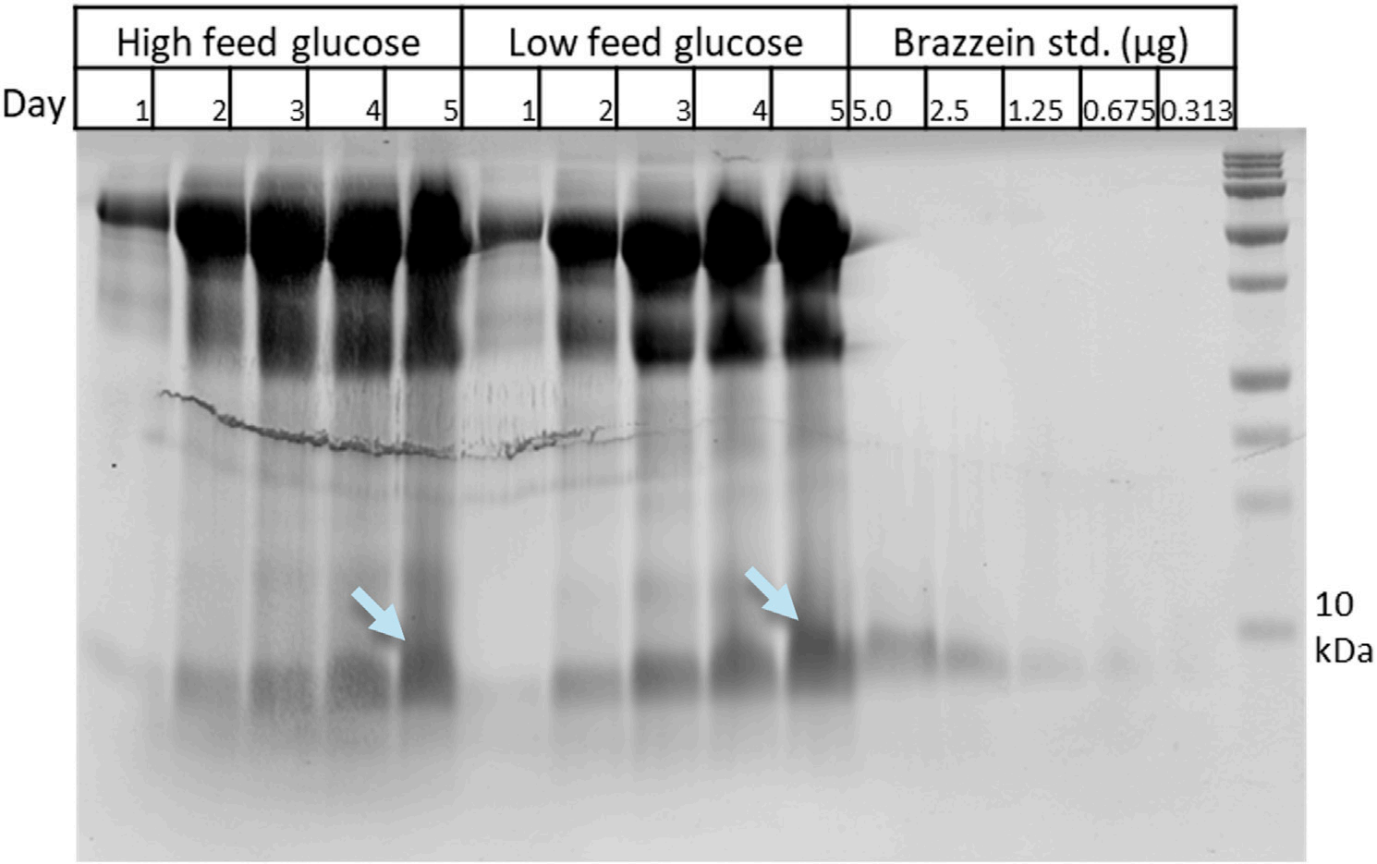
SDS-PAGE analysis of the Ambr bioreactor of strain M3730 (pH 4.8) samples from various timepoints (days 1–5) and a brazzein standard dilution series used for quantification using densitometry. Bands marked by light blue arrows were cut for MS analysis confirming the target protein brazzein (data not shown). Results from the Ambr bioreactor cultivation with strain M3728 (pH 4.0) provided very similar results.

Bioreactor cultivations were carried out in Ambr 250-mL reactors using low and high glucose feeding and pH values for M3728 (pH 4.0) and M3730 (pH 4.8). Supernatant samples were collected daily (day 1–5). The brazzein production levels were quantified with SDS-PAGE densitometry against a brazzein standard (Figure 3). Based on the densitometry analysis, the production in both low and high glucose feed was approximately 1.3 g/L. Broths from both reactors were pooled, and the product was enriched by ultrafiltration in deionized water (Supplementary Figure S4), followed by UPLC-MS to assess purity. We have not analyzed the intracellular fraction of the brazzein production strains; however, since the separation of the secreted protein from its cellular biomass occurs under very gentle conditions, cells very rarely undergo lysis during this step in downstream processing (data not shown).

NanoDSF thermal scans (350/330 nm ratio vs. temperature) did not yield typical S-shaped melting curves that are observed for most proteins (Gao et al., 2020), (Supplementary Figures S5, S6). However, in the first derivative of the curves at 330 nm or 350 nm, a peak at 64 °C could be detected. In the nanoDSF scattering measurement, the sample at the original concentration was aggregated after increasing the temperature to 72 °C. Overall, the results were indicative of a thermostable protein. However, due to the limitations of the method, the T_m_ is likely not representative. Therefore, other techniques were used to evaluate if brazzein was properly folded and to analyze its stability (i.e., CD spectroscopy, Timegated™ Raman spectroscopy, and LC-HCMS spectroscopy).

**FIGURE 4:**
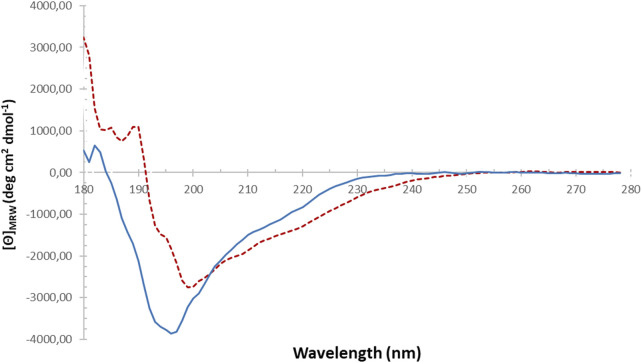
Circular dichroism spectra of the purified brazzein expressed in *T. reesei* at 22 °C (blue solid line) and 94 °C (red dashed line).

The overall secondary structures of brazzein can be observed from the CD spectra at 20 °C, and its thermostability, attributed to the retention of most of its main secondary structure, is demonstrated at 94 °C (Figure 4). Elucidation of the spectra reveals a mix of α-helical and anti-parallel β-sheets, which are comparable to X-ray and in-solution NMR structures (Table 2). Therefore, brazzein produced by *T. reesei* consists of a balanced mix of α-helical, β-sheet structures and loops.

**FIGURE 5:**
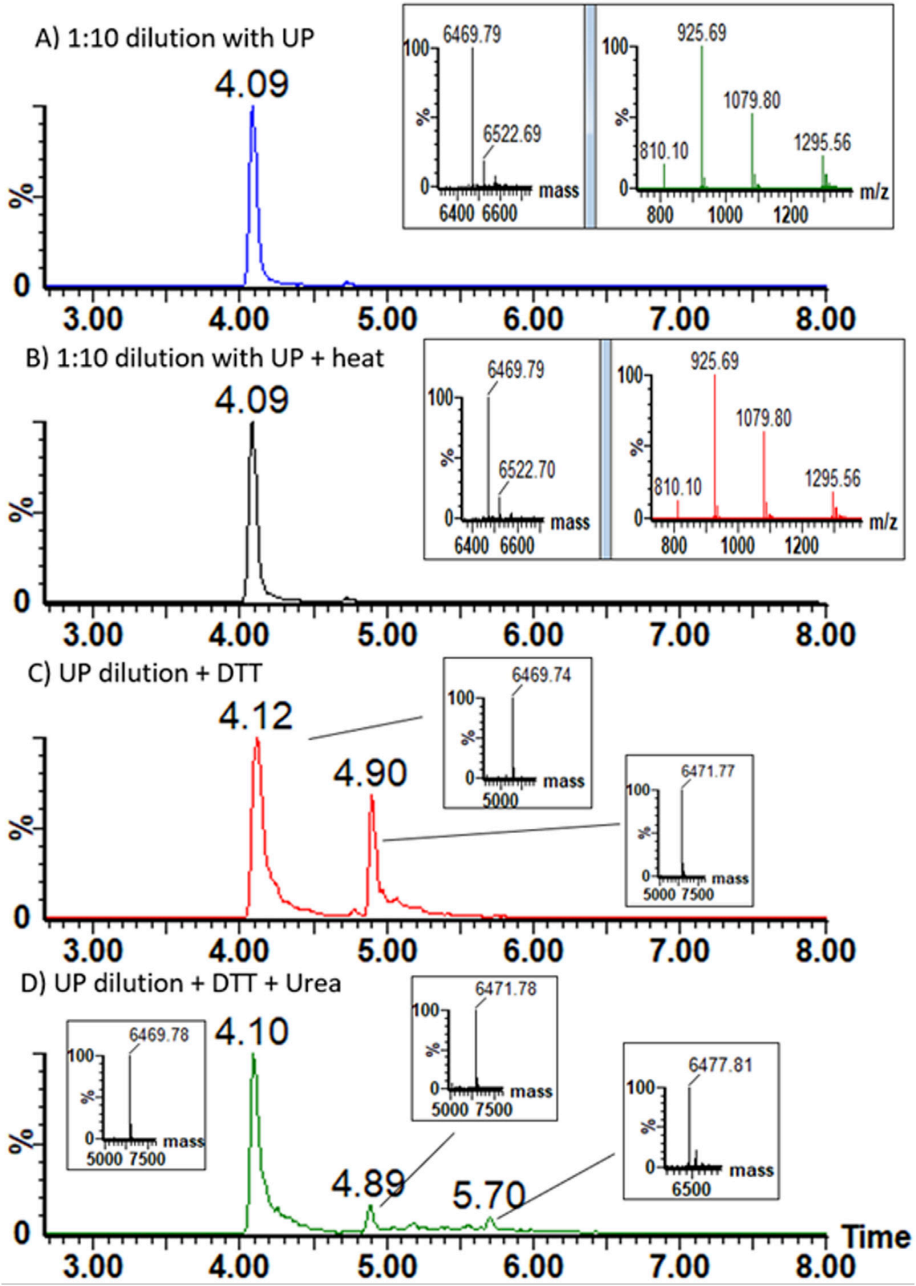
Chromatograms of the **(A)** non-reduced sample, **(B)** heat-treated sample, **(C)** DTT-reduced sample, and **(D)** DTT sample with urea dilution. Chromatograms are mass-filtered for clarity (m/z 926.3 ± 1 Da); insets display MaxEnt 3 deconvolution results and mass spectrum in **(A,B)** and the deconvolution results in **(C,D)**. Deconvolution results from different elution times show the non-reduced mass with RT 4.1 min, one reduced disulfide bridge (+2 Da) with RT 4.9 min **(C,D)** and the deconvoluted mass of fully reduced protein with RT 5.7 min in **(D)** (+8 Da). Note that MaxEnt 3 shows deconvolution results as M + H.

Liquid chromatography (UPLC-MS) followed by high-resolution mass spectroscopy (LC-HCMS) of purified brazzein samples confirmed the correct amino acid sequence. The observed mass of the non-reduced sample was 6,468.79 Da, matching the expected mass with 5 ppm accuracy with an N-terminal pyroglutamic acid Gln (−17.027 Da) modification (Figure 5A), while heat treatment had no effect (Figure 5B) forms, with and without N-terminal pyroglutamic acid, and N-terminal pyroglutamic acid formation is common during recombinant protein production (Shih et al., 2014). When reduced with DTT, brazzein showed notable resistance to reduction, particularly considering the small size of the protein. Subsequently, after 30 min of reduction at 50 °C, 15 mM DTT produced a pronounced appearance of a partly reduced variant in which only one disulfide bridge was reduced. Fully reduced species could only be observed after dilution of the reduced sample with saturated urea (Figures 5C,D).

Timegated™ Raman spectroscopy of brazzein showed the typical spectrum of an α-helical/β-sheet protein that contains disulfide bridges (Figure 4). In Table 1, the bonds are assigned based on Supplementary Table S3 and additional references. The Fermi doublet of tyrosine at 850/830 cm^−1^ of 0.77 showed that the tyrosines (11.1% of brazzein) are in an ionized state (Siamwiza et al., 1975), indicating that some phenol rings are involved in hydrophobic-type interactions. This ratio likely reflects the heterogeneous nature of tyrosines within the brazzein protein at this neutral pH (Hernández et al., 2016). The peak at 725 cm^−1^ is ot particular interest, which cannot be assigned easily to the most common protein peaks. However, when comparing this peak with the LC-HCMS results (pyroglutamic acid Gln), the peak can be confidently assigned to the γ(C=O) amide bond in L-pyroglutamic acid (Wagner and Baran, 2004). Additional evidence is the fairly uncommon protein Raman peak at 1,765 cm^−1^ and the shoulder in the amide I peak at 1,622 cm^−1^. Tyrosine shows a Raman band near 635 cm^−1^; however, the relatively high lysine content (13.3%) could also contribute to this region. The disulfide bonds are evident from the peaks at Raman shifts of 468 and 550 cm^−1^. Brazzein does not contain tryptophan, and therefore the strong peak at approximately 880 cm^−1^ is likely due to glutamate (7.4% of brazzein). We observed typical protein backbone and phenylalanine peaks between 910 and 1,210 cm^−1^ (Rygula et al., 2013). The amide III peak shows a maximum due to α-helices; however, as seen by the shoulder of the peak at 1,317 cm^−1^ (changes in the backbone), the amide II peak shows its maximum due to both α-helices and β-sheet structures (Rygula et al., 2013). The small peaks at 1,525 cm^−1^, which are likely -C=C- stretch of either histidine or tyrosine, are not common in protein samples. Most often, these peaks are assigned to a carotene type of compound present as either media components or cell debris. However, the other strong peak of the carotene fingerprint at 1,155 cm^−1^–1,160 cm^−1^ is missing (Tschirner et al., 2009). The peak at 1,564 cm^−1^ can be assigned to lysine (13% of brazzein), even though histidine (1% of brazzein) and glycine (1% of brazzein) also have a weak peak at this region. The amide I region shows some unique features. The smoothed peak shows a maximum at approximately 1,450 cm^−1^, indicative for α-helical proteins (Rygula et al., 2013). However, due to the N-terminal pyroglutamic acid Gln, the shoulder at 1,622 cm^−1^ can be assigned to the ν(C=O) amide bond (Wagner and Baran, 2004). The shoulder at 1,658 cm^−1^ can be assigned to β-turns and amide I (β-sheet) (Rygula et al., 2013; Sadat and Joye, 2020). Another non-typical protein Raman peak was observed at 1,765 cm^−1^, which can be assigned to the ν(C=O) carboxylate bond of pyroglutamic acid Gln. The peak at 1,884 cm^−1^ could be a possible overtone of arginine (3.7% of brazzein).

**TABLE 1:** Identification of Raman peaks and the corresponding bonds in brazzein.

Peaks at wavenumber [cm^-1^]	Bond type	Remarks
410	O vibration	Likely media component (e.g., PO_4_); could also be a weak Arg peak (De Gelder et al., 2007)
468–475	S–S stretching	Disulfate bonds
546–550	S–S stretching vibrations	Disulfide bonds
635	Tyr n-plane ring bending motions of His	De Gelder et al. (2007) Koenig and Sutton (1970)
727	Pyroglutamic acid	Wagner and Baran (2004)
817	Tyr	De Gelder et al. (2007)
880	Glu	De Gelder et al. (2007)
910	Pro	De Gelder et al. (2007)
996	N–Cα–N	Likely changes in the backbone
1,030	Phe	
1,081	Phe	
1,207	C–N (or Tyr and Phe)	De Gelder et al. (2007)
1,245	Amide III (α helix)	
1,320	C_α-H_	Likely changes in the backbone
1,410	C–H (general)	
1,525	C=C stretching	
1,564	Lys (His and Gly show weaker signal)	Zhu et al. (2011)
1,622	ν(C=O) amide	Wagner and Baran (2004)
1,640–1,655	Amide I (α-helix)	
1,668	β-turn and amide I (β-sheet)	Sadat and Joye (2020); Rygula et al. (2013)
1,765	ν(C=O) carboxylate	Wagner and Baran (2004)
1,890	Possible Arg overtone	
1,984	?	

^a^ Typically, 880 cm-1 is assigned to Trp; however, brazzein does not contain tryptophan. Glutamate can produce a strong signal at this wavenumber (7.4% of brazzein is glutamate).

**FIGURE 6:**
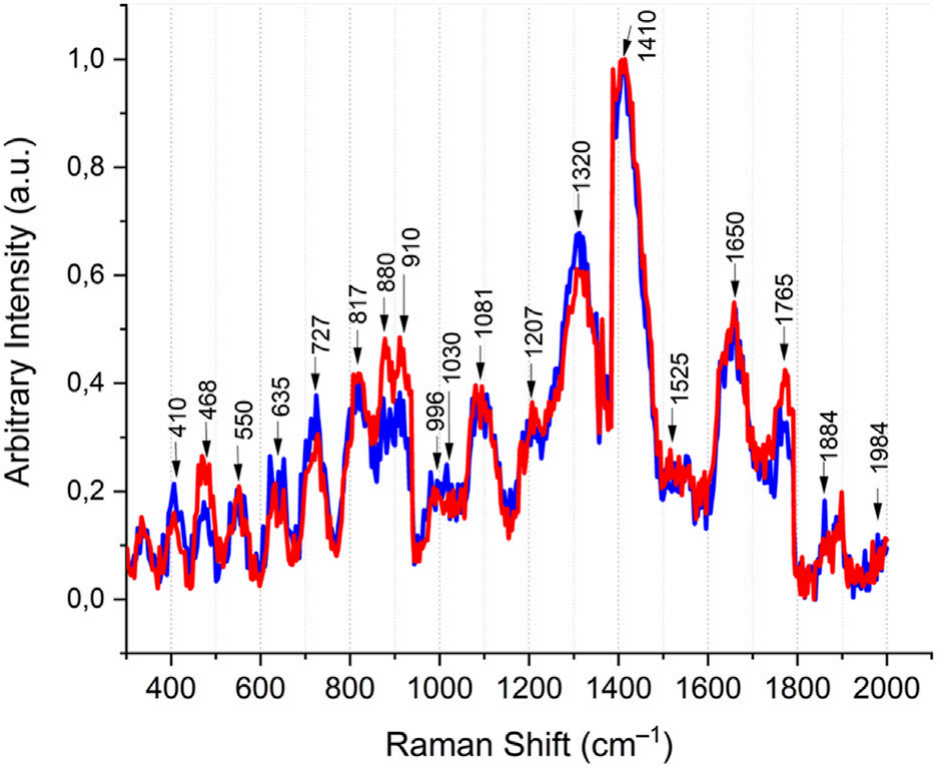
Timegated™ Raman spectrum of brazzein (blue: 20 °C; red: 75 °C). The spectrum is normalized and baseline-corrected. The spectra were not smoothed for a more detailed comparison. The major peaks indicated by black arrows are identified in detail in Table 1.

The distinct Raman spectrum of brazzein at room temperature or after heating for 20 min at 75 °C showed only minor changes (Figure 6), indicative of an intact secondary structure. In addition, though the spectrum was normalized and baseline-corrected, the raw data showed no loss in signal intensity (data not shown), indicating that brazzein was not aggregated (i.e., no protein was lost during centrifugation after heating). The minor changes were observed in the proline peak at 910 cm^−1^–911 cm^−1^, the glutamate peak at 877 cm^−1^–880 cm^−1^, and an S–S peak at 468 cm^−1^.

**FIGURE 7:**
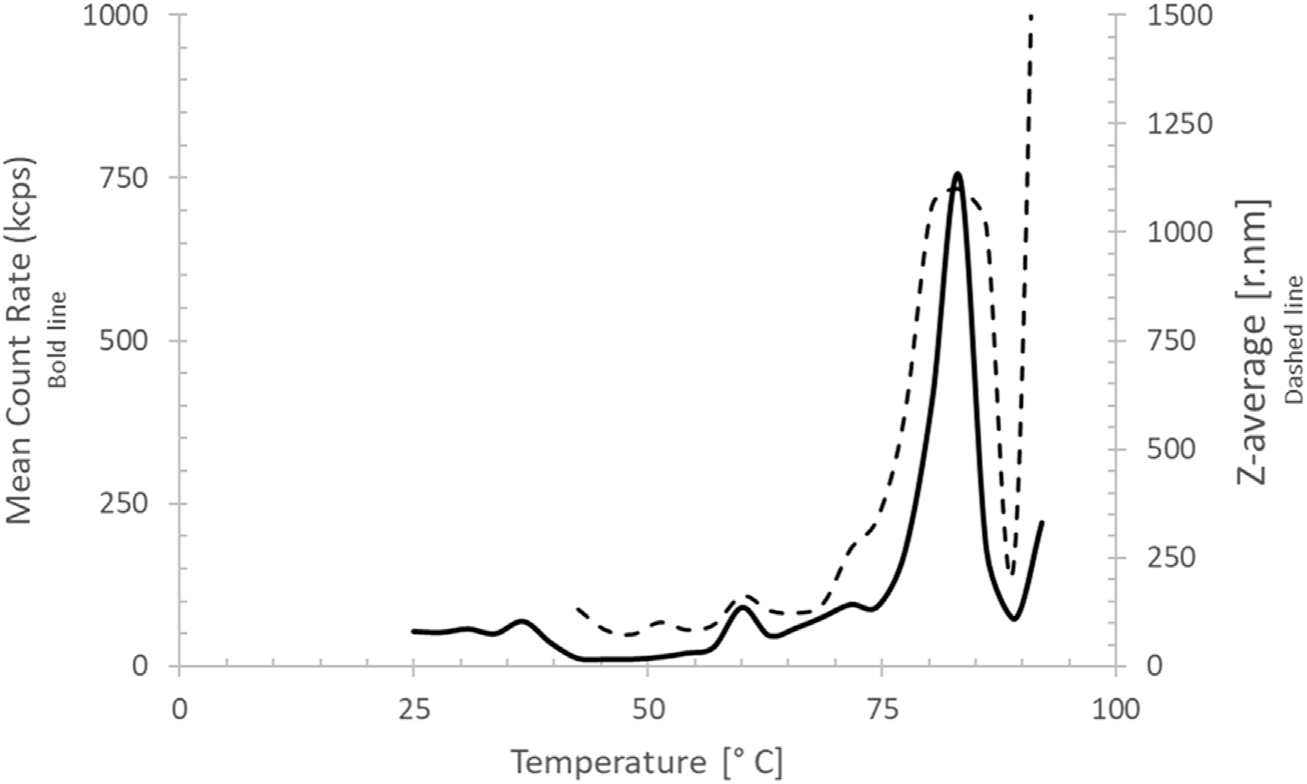
Dynamic light scattering thermal scan of brazzein. The mean count rate (in counts per second) is depicted as a bold line, and the z-average (radius in nm) is depicted as a dotted line. Brazzein aggregates fully at approximately 80 °C.

DLS of the purified brazzein sample that was stored at 4°–8 °C for 24 months showed that 99.6% of brazzein was still in soluble monomeric conformation with a radius of 1.9 nm at room temperature. A small impurity (0.4% w/w) was also observed (Supplementary Figure S6), which is likely present due to aggregates. Incremental heating of the sample, while measuring the DLS, indicated that the start of aggregation was at approximately 76 °C, verifying the high thermostability of brazzein (Figure 7) after long-term storage.

## 4 Discussion

Filamentous fungi are traditionally used in the bioprocess industry for the large-scale production of industrial enzymes. Several new approaches for producing food ingredients are also emerging. The filamentous fungus *T. reesei* is an important host for the production of heterologous proteins, as it is capable of secreting large amounts of hydrolytic enzymes, as high as up to 100 g/L of protein under optimal conditions (Cherry and Fidantsef, 2003). In our laboratory, *T. reesei* has also been used for the production of biopharmaceutical and food proteins (Landowski et al., 2016; Aro et al., 2023) and a single heterologous protein at yields exceeding 80 g/L (VTT, unpublished data). In this study, we used *T. reesei* in the production of sweet-tasting protein brazzein. Using the SES-system (Rantasalo et al., 2019), it was possible to produce brazzein at 1.3 g/L in a 250-mL bioreactor using 100 mL of glucose-containing medium. Although the expression levels of the strain with the expression cassette containing the *cbh1* promoter were not confirmed in bioreactor cultivations over 100 mL, the levels in 24-well plate cultures were in the same range as those of the SES-system. Based on earlier results of VTT in collaboration with industrial partners (data not shown), brazzein strains are very likely productive at similar levels at larger scales. However, proper scaling-up experiments should be carried out to ensure this. Compared to previous reports (Berlec et al., 2006; Lamphear et al., 2005; Lee et al., 2019; Poirier et al., 2012), the production levels described in this study for *T. reesei* are several folds higher than those for *E. coli* or yeast systems.

The CD spectra of brazzein expressed in *T. reesei*, as shown in Figure 4, show the differences between ambient temperature and above 90 °C; however, deconvolution of the spectra shows no differences. We suspect that the additional smoothing of the analysis software (BeStSel v1.3.230210) or insensitivity of our CD spectroscope resulted in misclassifying the loop and coil structures. However, when comparing our CD spectra to earlier brazzein studies (Liu et al., 2021), the CD spectrum of brazzein at 22 °C aligns well with that of the wild-type produced in *E. coli*. Brazzein produced by Liu and co-workers lacks the N-terminal pyroglutamic acid Gln, and differences in the CD spectra are observed between 205 and 225 nm, indicating slight differences in β-sheet and/or random coil structures. When overlaying the CD spectra reported here (Figure 4) with the data of Liu et al. (2021), the spectra between 190–205 nm and 215–25 nm were identical at ambient temperature (data not shown). When comparing both CD spectra with those of brazzein expressed in *Nicotiana tabacum* cv. Xanthi, which also lacks the N-terminal pyroglutamic acid Gln (Choi et al., 2022), it becomes apparent that the α-helical structures of brazzein vary widely depending on the conditions of production, purification, and storage (Table 2).

**TABLE 2:** Secondary structure analysis of the recombinant brazzein and its mutants.

Sample	22 °C^a^	94 °C^a^	4HE7^b^	2KGQ^c^	*N. tabacum* 25 °C^d^	*E. coli* 25 °C^e^
Helix	48.3	48.3	33.3	30.2	9.9	4.9
Antiparallel β-strands	48.3	48.3	27.8	26.4	19.7	26.8
Parallel β-strands	0.0	0.0	-	-	?	2.2
Turn	0.0	0.0	-	-	?	20
Other	3.4	3.4	-	-	?	43.9
Total	97.8	100	-	-	-	97.8

^a^ Results were analyzed using BeStSel v1.3.230210. Brazzein in H_2_O.

^b^ Structure of brazzein determined with X-ray crystallography at 20 °C and pH 4.0 in 0.9–1.0 M sodium citrate buffer (Nagata et al., 2013).

^c^ Structure of des-pGlu brazzein determined with in-solution NMR at 37 °C and pH 5.2 in 90% H_2_O/10% D_2_O at an ionic strength of 20 and ambient pressure (Cornilescu et al., 2009).

^d^ Brazzein lacking the N-terminal pyroglutamic acid Gln (Choi et al., 2022).

^e^ Brazzein lacking the N-terminal pyroglutamic acid Gln (Liu et al., 2021).

**FIGURE 8:**
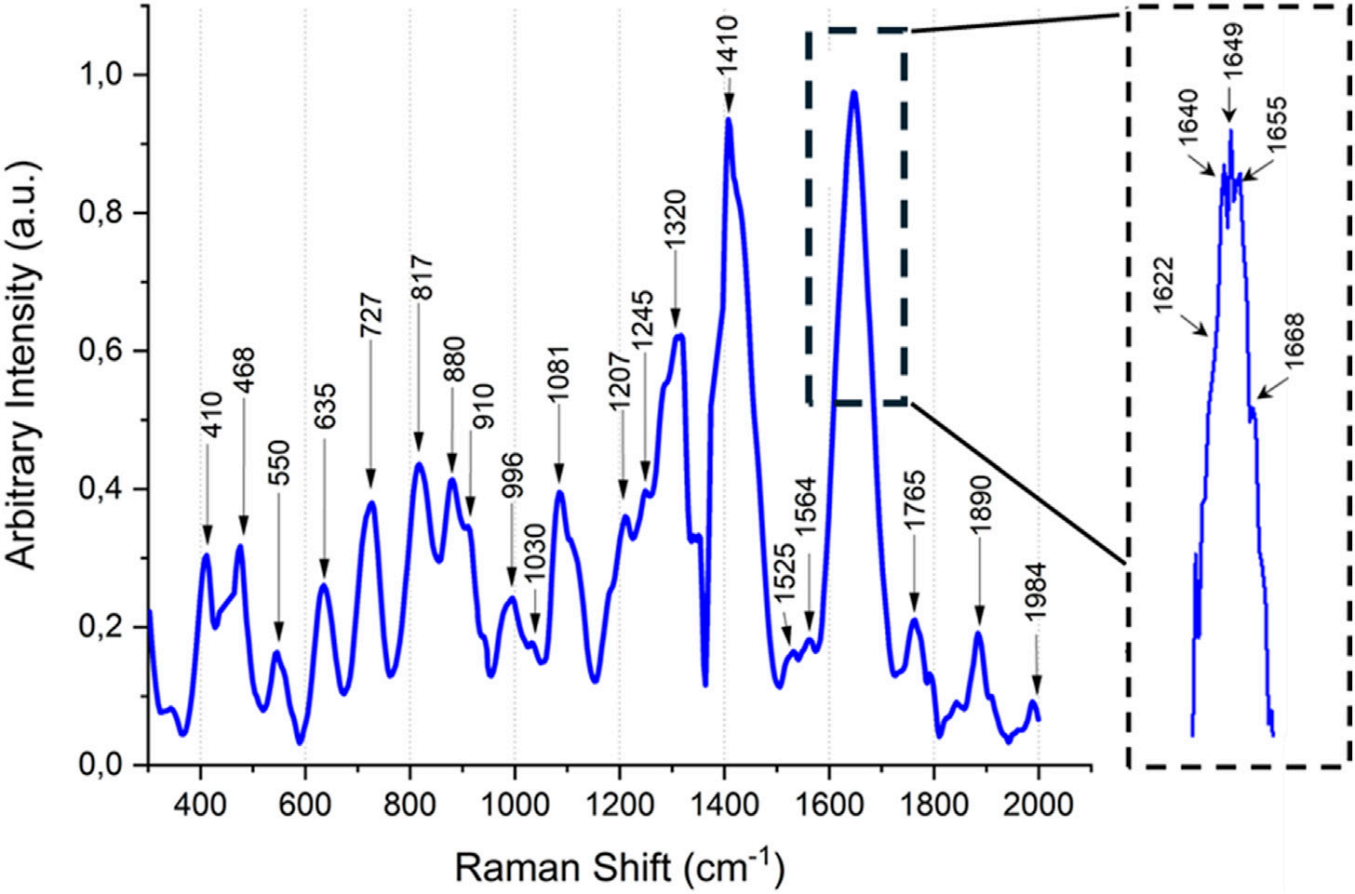
Timegated™ Raman spectrum of brazzein. The spectrum is normalized and baseline-corrected. The major peaks indicated by black arrows are identified in detail in Table 1. Inset: the amide I peak providing more details.

Analysis of the Raman spectra of brazzein revealed that its secondary structure was similar to that of previously determined Timegated™ structural models (Nagata et al., 2013). Brazzein expressed in *T. reesei* had four disulfide bonds, as determined by LC-HCMS (see Figures 5A–D), contained an N-terminal pyroglutamic acid (see Figures 5, 8), and displayed α-helical and β-sheet structures (Figure 8), which is in line with earlier reports (Nagata et al., 2013). The presence of N-terminal pyroglutamic acid was further confirmed by the Raman spectra through the detection of the ν(C=O) carboxylate bond of pyroglutamic acid Gln. In addition, when comparing models of the amide I peak (Sadat and Joye, 2020) to our Timegated™ Raman spectra, we observed the typical maxima of α-helixes and β-strands even though the spectra were noisy. The overall reason for this is the relatively low concentration of brazzein in the solution and the residual fluorescence background. In addition, the noisiness of the spectra is masked by fluorescence. The PicoRaman M2 instrument allows selection of spectra in the temporal domain. Here, we used separation of binned photons containing the most Raman information of the sample (i.e., separating the part of the spectrum with the most auto-fluorescence), however some small residual fluorescence may remain. The post-processing performed using OriginPro software includes baseline correction and some minor spectral smoothing. Despite the noisiness of the spectra, they are consistent since we carried out several repetitions with one sample, as shown in Supplementary Figure S8.

Brazzein is a thermostable protein, which was confirmed by CD spectroscopy (Figure 4; Table 2) and by comparing Timegated™ Raman spectra after heating brazzein for 20 min in deionized water at 75 °C to Timegated™ Raman spectra at room temperature (see Figure 6). On the other hand, the nanoDSF measurements (Supplementary Figures S4, S5) highlight the limitations of this fluorescence method to detect thermostability of brazzein. Since brazzein is a small protein and does not contain any tryptophan residues, significant fluorescence changes might not be observed, making it challenging to detect unfolding.

To evaluate the secondary structure of brazzein, a non-invasive technique without labels showed, as reported earlier (Zuo et al., 2024), that the α-helical structure was partly lost at 94 °C compared to that in the room temperature in the CD-spectra. Timegated™ Raman spectroscopy at two different temperatures did not indicate any major changes in the secondary structure of brazzein. These changes in the glutamate peak (880 cm^−1^) are likely due to backbone effects, since of the four glutamate residues, three are located in the loop regions. Only Glu35 resides at the first residue of the β-sheet structure. This further supports the slight reduction in the signal around the C_α-H_ peak at 1,317 cm^−1^ in the Timegated™ Raman spectra due to heating (Figure 6). Intact protein mass spectroscopy, however, detected changes in the proline-associated region. Brazzein has only one proline, Pro11, which sits at the start of the α-helix, and as concluded by Zuo et al. (2024), the disulfide pair Cys16–Cys37 plays an important role in thermal stability and sweetness activity.

The intact mass spectroscopy experiments demonstrated that brazzein retains identical chromatographic and mass spectrometric responses after heating at 70 °C for 20 min, indicating its relatively high resistance to reduction. The reduction experiments suggest that one of the four disulfide bonds is more accessible to reduction, whereas a fully reduced protein could be observed only after denaturation with urea. The absence of a fully reduced brazzein signal from the aqueous (non-denatured) sample could also be due to aggregation of brazzein that lacks disulfide bonds.

Brazzein combines water solubility, small size, and strong non-covalent binding to the sweet-taste receptor with exceptional heat stability. This combination of features requires a stable three-dimensional structure, in which the dense disulfide bonding likely plays a key role. The apparent importance of disulfide bonding in stabilizing brazzein is supported by a previous study (Ghanavatian et al., 2016), in which long reduction times were used to establish the low stability of reduced brazzein in different urea concentrations. In addition, correct folding facilitated by the disulfide bonds is crucial for the perception of the sweetness of brazzein (Zuo et al., 2024). Moreover, protein stability was assessed with DLS thermo-scanning to detect aggregation in a 24-month-old brazzein sample (stored in deionized water at 4 °C–8 °C) aggregated at 80 °C, confirming the stability of brazzein produced by *T. reesei*.

Earlier reports showed that mutations causing disruptions to the loop of amino acids 9–19, general protein misfolding, and the elimination of disulfide bonds, especially the terminal disulfide bond, significantly reduce sweetness and receptor binding (Assadi-Porter et al., 2010; Singarapu et al., 2016; Dittli et al., 2011). Thus, we speculate that brazzein produced with *T. reesei* retains its sweetness since we demonstrated correct folding, including correct disulfide bond formation. However, we have not confirmed this with panel-based taste testing.

## 5 Conclusion

We demonstrated that brazzein with correct folding can be produced using the filamentous fungus *T. reesei*. LC-HCMS in combination with Timegated^TM^ Raman spectroscopy outperformed other previously implemented approaches for analyzing the folding of small proteins that lack the amino acid tryptophan, a feature that is critical for nanoDSF measurements. Since correct folding is essential for brazzein to interact with its receptors on the tongue, the approaches presented here, along with previously discussed methods (Saraiva et al., 2023), could provide an alternative when taste testing cannot be performed.

Based on our earlier cost models for heterologous protein production using *T. reesei* (Järviö et al., 2021) and extrapolating production costs for secreted protein as described earlier (Taiwo et al., 2022; de Lima et al., 2022), we can estimate 57 €–80 € per kg of brazzein at 1.3 g/L production levels, which is equivalent to 29 €–40 € per ton, when normalized for the sweetness of sucrose (i.e., cost per ton divided by 2000). Compared to 406 € per ton of sucrose at the time of writing^1^, this indicates a significant reduction of cost for similar sweetness levels. For comparison, several patents have reported industrially relevant yields (e.g., in yeast: 0.3 g/L (HYEON, 2022) or in *Aspergillus oryzae* producing brazzein at a high yield in a functional form (Vind et al., 2011)). However, optimized strains (Aro, 2025) with higher production levels could reduce the cost to 20 € per kg or even lower. Therefore, based on the results of this study, it is possible to achieve the production of brazzein with correct folding at industrially and economically relevant levels using *T*. *reesei*.

## Data Availability

The original contributions presented in the study are included in the article/Supplementary Material, further inquiries can be directed to the corresponding author.

## Footnotes

1 Source: https://www.isosugar.org; data from 15 August 2025.
