# Supplementary material for: Label-free analytical characterization of brazzein produced with the filamentous fungus Trichoderma reesei
Source: Front Bioeng Biotechnol. 2025 Dec 12;13:1688495. doi: 10.3389/fbioe.2025.1688495 (PMC12741742; doi:10.3389/fbioe.2025.1688495)
Supplement: Supplementary file 1 [file Supplementaryfile1.docx]

Supplementary Material

Label-free Analytical Characterization of Brazzein Produced with the Filamentous Fungus *Trichoderma reesei*

**Dominik Mojzita^1^, Nina Aro^1^, Juha Kontturi^1^, Martin Kögler^2^, Emilia Nordlund^1^, Waltteri Hosia^1^, Marco G. Casteleijn^1^***

1. VTT Technical Research Centre Finland, Espoo, Finland.
2. VTT Technical Research Centre Finland, Oulu, Finland.

*** Correspondence:**Corresponding Author
marco.casteleijn@vtt.fi

Keywords: Brazzein, Raman Spectroscopy, Mass Spectroscopy, Fungi, Sweet Protein

| 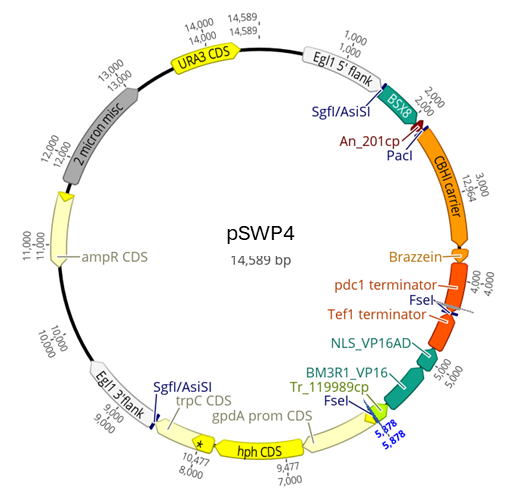 | 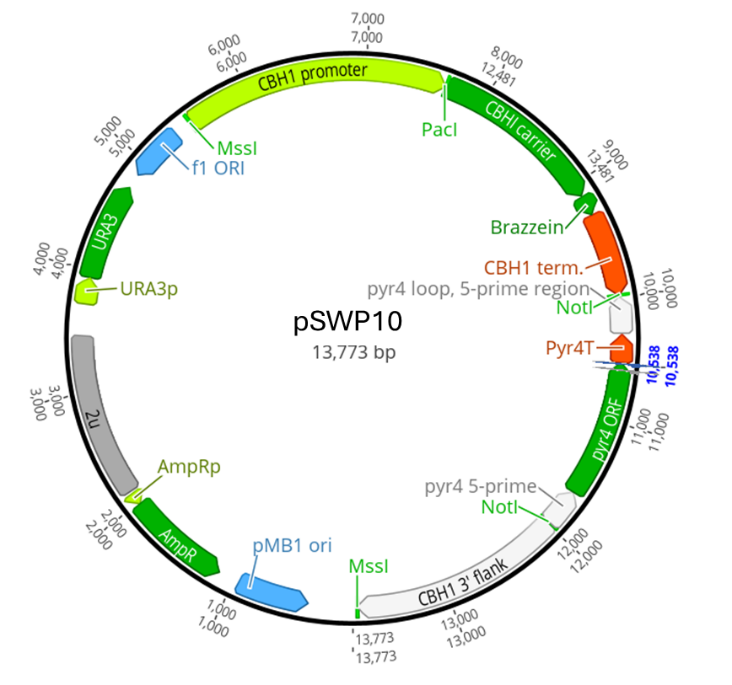 |
| --- | --- |
| **Figure S1.** Plasmid maps of pSWP4 and pSWP10. The plasmid map of pSWP3 is identical to PSWP4, with the exception of the CBHI carrier DNA element where the hydrophobic signal sequence is inserted instead. | |

# Table S1. Expression cassette elements

| Plasmid | Promoter | Signal sequence | Fusion partner | Protein of interest | Selection marker | Target Loci |
| --- | --- | --- | --- | --- | --- | --- |
| pSWP3 | SES – BSX8 | Hydrophobin | - | brazzein | Hygromycin | Egl1 |
| pSWP4 | SES | - | CBHI | brazzein | Hygromycin | Egl1 |
| pSWP10 | CBHI | - | CBHI | brazzein | Pyr4 | CBHI |

# Table S2. Guide RNA and PCR screening plasmids

| **Name of primer** | direction | DNA sequence |
| --- | --- | --- |
| crRNA Egl1 | forward reverse | \| TATCCTGGAGGGCAACTCGAGGG \| \| --- \| \| GCGAGTAGTCTGTATTTTAGCGG \| |
| crRNA CBHI | forward reverse | \| CTACTTCGTGTCCATGGACG \| \| --- \| \| TATTGGCTACAGCGGCCCCA \| |
| Egl1 PCR screening primers | 5’ forward  5’ reverse  3’ forward  3’ reverse  ORF forward  ORF reverse | \| GGAAGAGTACGCTCAGAGG \| \| --- \|   TCAGTACTCACAATAATCAC  GCAAGCGCCAAGACAAGTGC  GAGTGCTGGTCGCCGGAGAA  ATGGCGCCCTCAGTTACAC  AGTCGTTGCTATACTGGCAC |
| CBHI PCR screening primers | 5’ forward  5’ reverse  3’ forward  3’ reverse  ORF forward  ORF reverse | AGCTCGGAGGTTTGTGACATG  TCAGTACTCACAATAATCAC  GCAAGCGCCAAGACAAGTGC  GGTTGACTTACTCCAGATCG  TGCCATGACTCACTGATTGG  CCAATACCGCCGCACTGGC |

# Table S3. Specific protein band assignments for different protein classes with a 532 nm laser; adapted from Rygula *et al.* (2013)(Rygula et al., 2013) and De Gelder *et al.* (De Gelder et al., 2007).

|  |  | **Wavenumber [cm^-1^]** | | | | | |
| --- | --- | --- | --- | --- | --- | --- | --- |
| **Bond type** | | **Alpha Helix** | | | **Beta sheet** | | **Alpha / Beta** |
| Amide I |  | 1657 | |  | 1669 |  | 1664 |
| Tyr,Trp,Phe |  | 1610 | |  | 1619 |  | 1621 |
| Indole ring (Trp) |  | | - |  | 1553 |  | - |
| C-H (def) |  | 1454 | |  | 1453 |  | 1482 |
| C-H (def) |  | - | |  | - |  | 1453 |
| Trp, Cα-H (def) |  | 1341 | |  | 1342 |  | - |
| Trp, Cα-H (def) |  | 1321 | |  | - |  | - |
| Amide II |  | 1273 | |  | 1236 |  | 1283 |
| Amide III |  | 1246 | |  | - |  | 1236 |
| C-N |  | 1130 | |  | 1123 |  | 1144 |
| Phe |  | 1036 | |  | 1036 |  | 1035 |
| Phe |  | 1009 | |  | 1007 |  | 1004 |
| N-Cα-C |  | 945 | |  | 945 |  | 935 |
| Trp |  | 880 | |  | 883 |  | - |
| Tyr |  | 856 | |  | 856 |  | 861 |
| Tyr |  | 832 | |  | 830 |  | 847 |
| Trp |  | 755 | |  | 764 |  | - |
| Tyr |  | 651 | |  | 647 |  | - |
| S-S |  | 512 | |  | 545 |  | - |

#
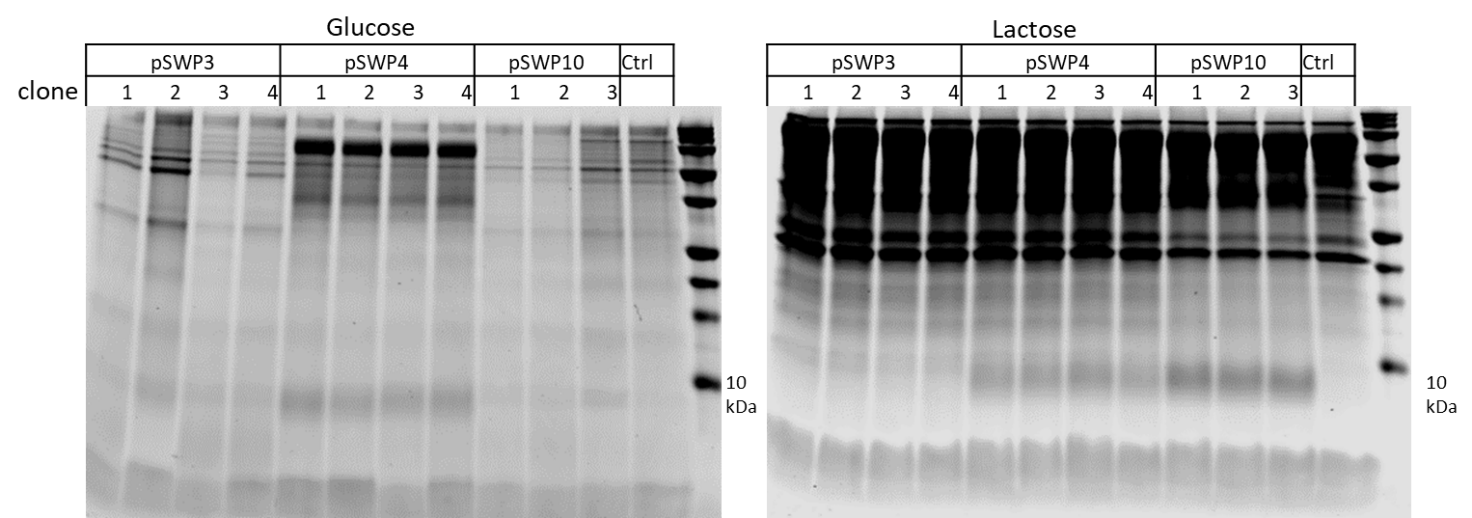


# Figure S2. SDS-page analysis of supernatants collected from the 24-well cultures at day 4. All the different expression cassettes (3-4 clones of each cassette) targeted to the egl1 locus showed putative brazzein production in the 24-well plate cultures when glucose containing media was used. The strongest brazzein bands were observed in the four SWP4 clones. When lactose medium was used as a growth medium, the strongest production was seen in the three SWP10 clones.

#
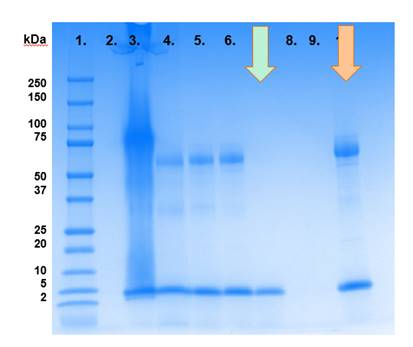


Brazzein

# Figure S3. Ultrafiltration of brazzein samples in deionized water. The sample indicated with the red arrow contains all secreted proteins, while the sample indicated with the green arrow is brazzein after purification. The brazzein band of 6.5 kDa is indicated with a black triangle. LC/MS analysis indicated that the sample is pure.

#
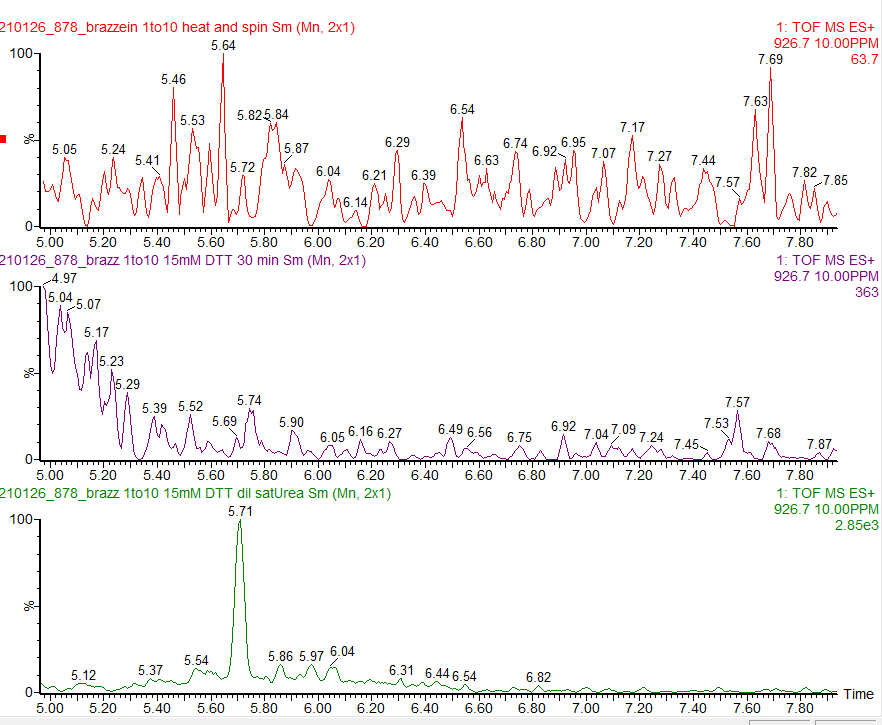


# Figure S4. Extracted ion chromatograms of 7+ charge state fully reduced protein, mass of 926.70 (10 ppm window). Upper = non-reduced sample, middle = reduced sample, bottom = reduced sample diluted 1:5 with saturated urea. Notably there is no observable signal in two upper ones.

#
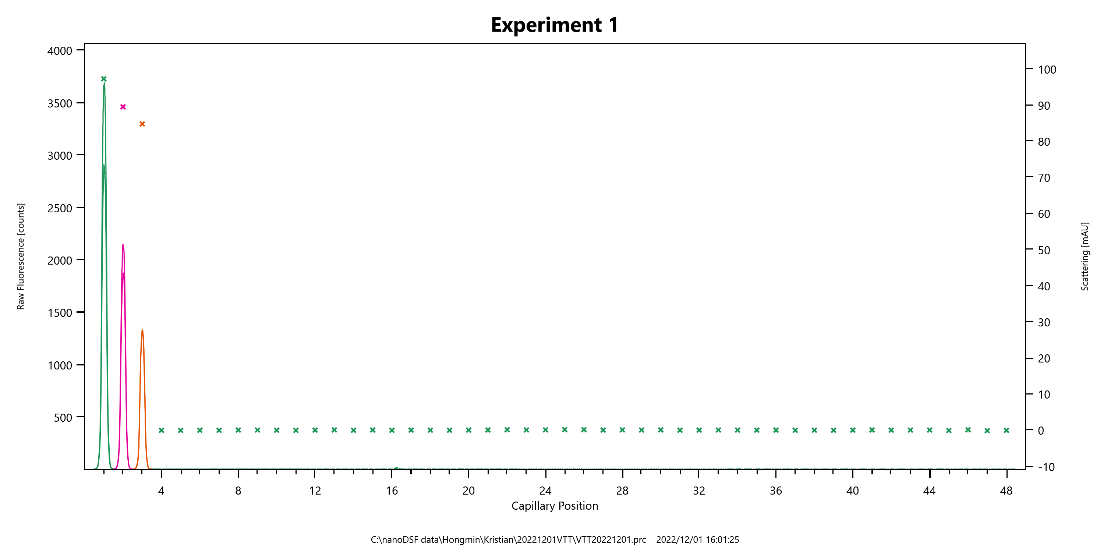


Scattering [mAU]

Capillary position

Raw fluorescence [counts]

# Figure S5. In pre-scanning (i.e. to optimize the fluorescence intensity) the excitation power of 100% is needed for detecting signals in a good range.

#
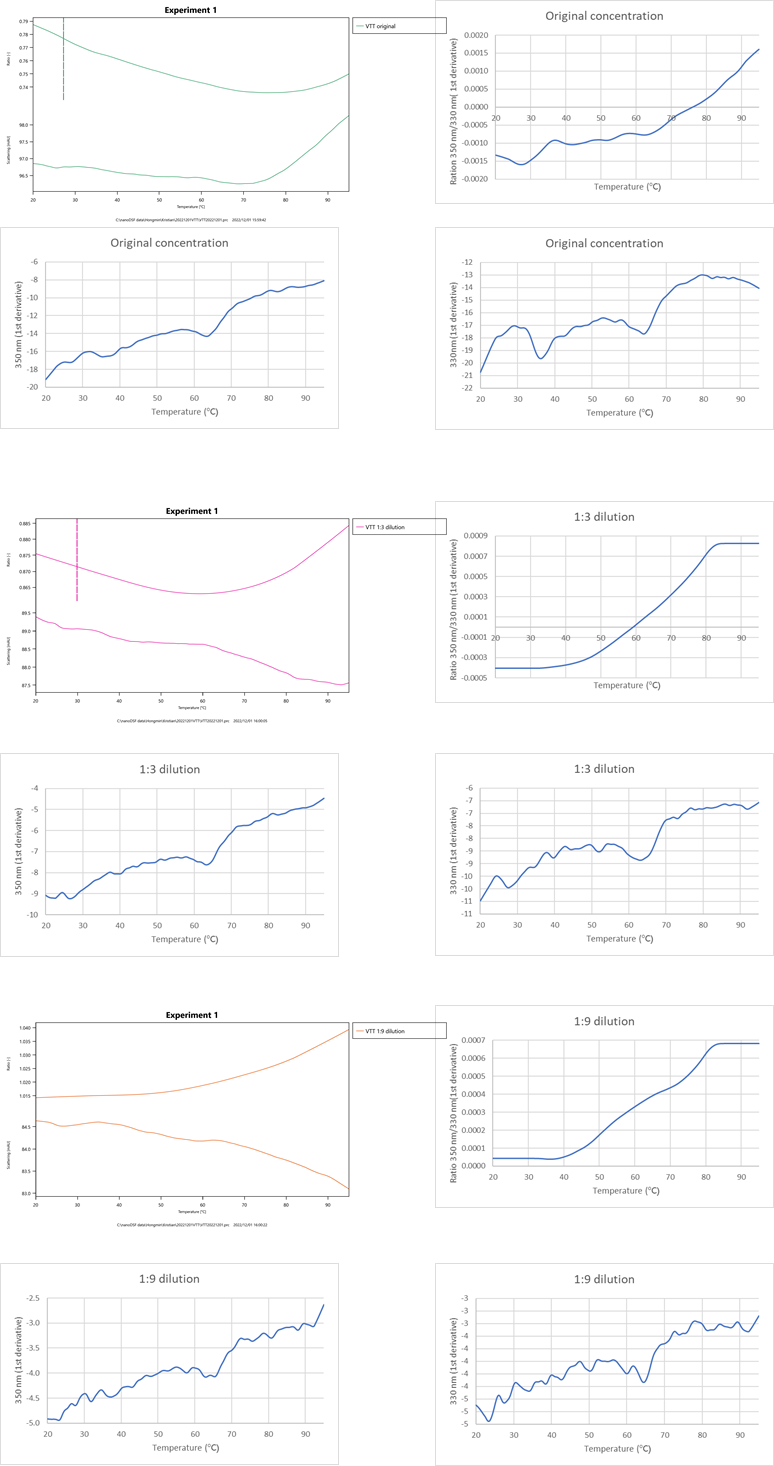


# Previous page: Figure S6. Melting scan and T_m_ analyses of the sample at three dilutions. Green = original concentration; magenta = 1:3 dilution: orange = 1:9 dilution.

#
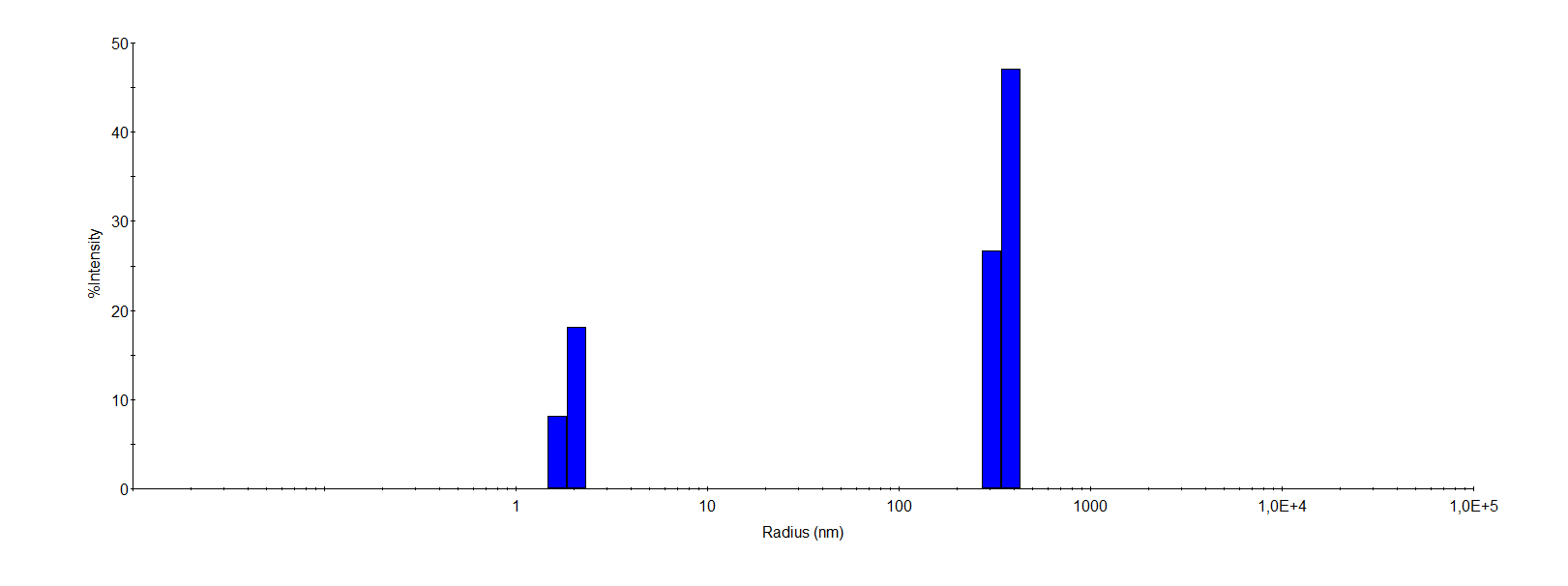


# Figure S7. Single DLS analysis with constant temperature, 20^o^ C.

#
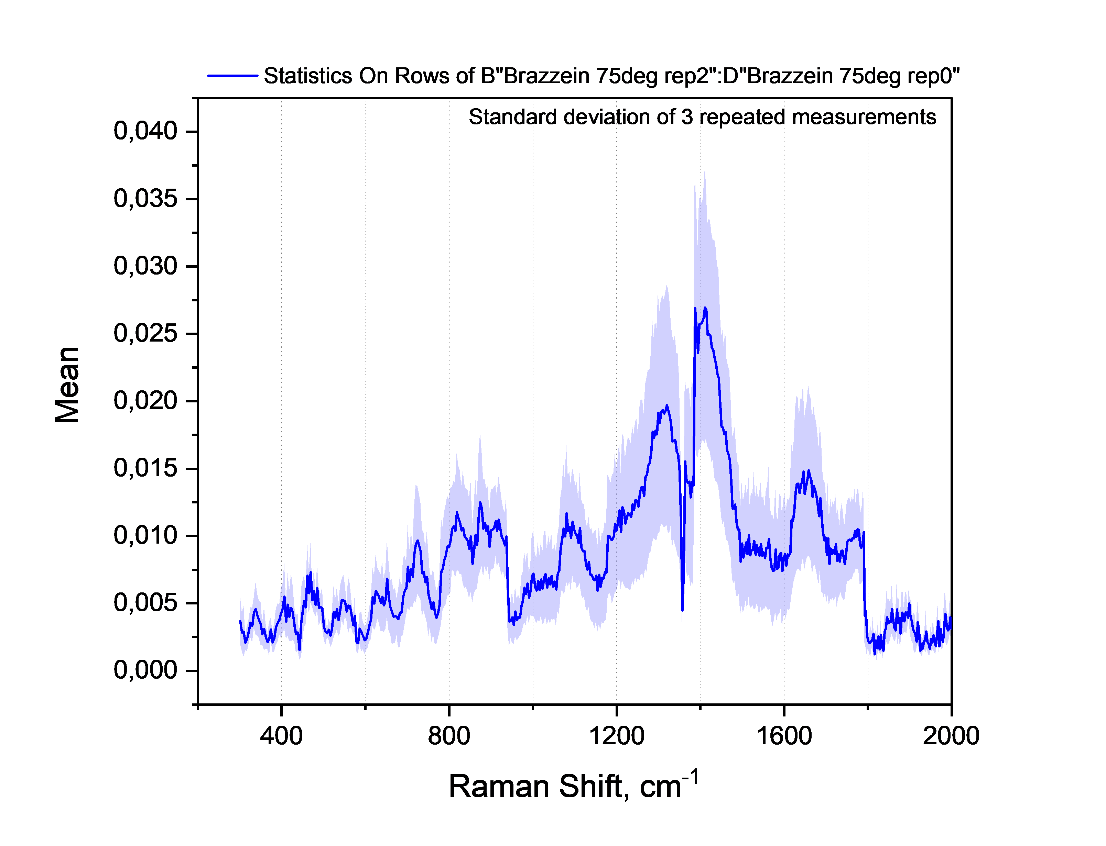


# Figure S8. Raw data indicating the variation of triplicate measurements in respect to the maxima TimeGated^TM^ Raman peaks.

# References

DE GELDER, J., DE GUSSEM, K., VANDENABEELE, P. & MOENS, L. 2007. Reference database of Raman spectra of biological molecules. *Journal of Raman Spectroscopy,* 38**,** 1133-1147.

RYGULA, A., MAJZNER, K., MARZEC, K. M., KACZOR, A., PILARCZYK, M. & BARANSKA, M. 2013. Raman spectroscopy of proteins: a review. *Journal of Raman Spectroscopy,* 44**,** 1061-1076.
